# The Medical Examiner, and American Medical Library; a Reply to Their Strictures

**Published:** 1840

**Authors:** 


ART. XV.—*The Medical Examiner.* Edited by J. B. BIDDLE, M. D., M. CLYMER, M. D., and W. W. GEHHARD, M. D., Philadelphia.

*The American Medical Library and Intelligencer. A concentrated record of Medical Science and Literature.* By ROBELEY DUNGLISON, M. D., M. A. P. S. Professor of the Institutes of Medicine and Materia Medica in Jefferson Medical College, Attending physician to Philadelphia Hospital, and Fellow of the College of Physicians of Philidelphia, &c. &c.

WE have no wish to come into collision, or to engage in any sort of controversy, with the conductors of these two interesting Periodicals. On the contrary, it is our earnest desire to preserve peace and live in harmony and good fellowship with all writers, as far as we can do so, consistently with the decision of our judgment, and the dictates of our conscience—but no farther. Neither jot nor tittle of what we believe to be true and useful will we sacrifice, in any case, on the alter of Concord ; because such an act, culpable in principle, and mischievous in tendency, is forbidden by the mandate of a superior Deity.

In our reviews of works of every description, however high and imposing may be their authorship, and whatever degree of favour they may have found elsewhere, we shall express our sentiments freely and fearlessly, without intending to give offence, but regardless of such offence as may be causelessly taken. Nor do we know any good reason why our Atlantic brethren should take exception at us for thinking differently from them respecting works, opinions, or doctrines in medicine, any more than why they should find fault with us, because we choose to reside on the west, while they prefer the east side of the Allegheny mountains. And we beg leave to assure them, that their dissatisfaction on either or each of these points will neither detract from the amount of our waking enjoyments, nor from the soundness of our repose. Worse still ; it will not even convince us that our opinions are groundless.

If they wish to reclaim us from error, they must condescend to *reason* with us. By dictation and censure they will gain nothing but our calm disregard.

These remarks have been drawn from us, by the manner in which our review of the late Professor Hosack's "Lectures," in the first number of this Journal, have been noticed by the two Journals, whose titles form the heading of this article. In this reference we allude more especially to the style and spirit of the notice contained in tne "American Medical Library and Intelligencer," which is neither courteous nor manly ; and which, as far as it has *any* meaning, "means mischief." But more of this presently.

To the notice contained in the "Medical Examiner," we have no material objection to offer, except that the doctrine maintained in it is erroneous and untenable, as we shall endeavour to make appear. The courtesy which marks it is such as ought to subsist, and always *will* subsist, between *cultivated* and *ingenuous* advocates of truth. It is the uncultivated, the disingenuous, or the ill-tempered, or the three united, that attempt to sustain their notions, or gratify their unkind feelings, by means which the soundness of science, and the comity of letters concur in disclaiming. One observation more before we commence our exposition of the fallacy of the doctrine contended for in the Examiner. Should a conflict ensue, on the ground of that doctrine, between that Journal and this (an event which it is our sincere desire to avert,) let it not be forgotton that the Examiner threw down the gauntlet, and that we only did not decline to lift it. The sentiment in question is one which we have long entertained, and often avowed. And on our last avowal of it, we expressed ourselves in a *general* way, without the slightest expectation or wish to excite a controversy. We repeat, therefore, and desire it to be borne in mind, that the Examiner is the assailant.

In our reeviw of Dr. Hosack's "Lectures," it will be remembered that we excepted to them chiefly on two grounds; their fearful hypertrophy of reference, quotation, and authority; and their incorrectness as a standard of *practice* for the *diseases of the West and South.* On their relation to the complaints of the Atlantic, middle and northern States we neither made then, nor mean to make now, a single remark. To decide on that point belongs to the practitioners of those States; and to their judgment the matter is referred.

To our censure of the quotation and-authority-stuffed style and manner of the "Lectures," the Editors of the Examiner do not object. On the contrary, we doubt not that, on that topic, they silently concur with us. The reason is plain. Their own style and manner, as well as the style and manner of the school, in which they have been educated, and of the authors they most admire, are entirely different. In plain terms, we think too favourably of their taste, to allow ourselves to hold it possible for them to approve of composition so utterly wanting in literary merit. But for the following sentiment, expressed in the Examiner, there is not the slighest foundation in nature, as the Editors would be themselves convinced, if, instead of fancying in their closets in Philadelphia, how things *may be* in the West, they would visit our Valley, and *learn* how they *are*. They would then perceive, in all its force, the truth of the Novelist's allegation, that a man rarely writes the worse, for having *some knowledge* of the subject he treats of.

"If," say the Editors, "the doctrine be carried out, that a student is to study medicine just in the spot where he is to practise it, our friends at Louisville will be narrowed down to a much more limited range of pupils, than the "Valley of the Mississippi. We apprehend that the same variety may be found between the diseases of different points of this locality, as between many of them and those of the seaboard."

That many people may call this remark *smart*, and some perhaps even *keen* and *shrewd*, we think probable. But no competent judge can possibly pronounce it either *sound* or *conclusive*. Worse still. It is not candid. Our reply to it shall be brief.

We have no where alleged that a "student is to study medicine *on the spot* where he is to practise it." Of this the Editors must be as sensible as we are. Why then have they virtually asserted the contrary? The answer is plain. They had nothing better to assert. They were bankrupts in solid matter for the support of their notion. They had therefore no alternative but to observe silence, and surrender at discretion, or deal in error—at least in a sophistical expedient no better than error. They remind us of the awkward and embarrassing condition of the Poet's Jack Skyscape, who, "Because he knew not what to say, he *swore.*" Because they knew not in what way to *reason*, they *played the sophist.*

The real substance of what we have said on the subject is, that medical pupils can be taught *correct* practice only in schools, whose professors have themselves a *practical* knowledge of the character both *general* and *special*, as well as of the *treatment* of the diseases, with which the pupils are to be afterwards concerned. And the position is, in itself, so intuitively clear, that we shall not attempt to illustrate or prove it. As soon would we labour to establish the fact, that ponderous bodies, when unsupported, fall to the ground.

But diseases are the product of the regions where they prevail. If the regions are alike therefore, however extensive they may be, the diseases will be also alike. This is another maxim in medicine, which no one versed in the profession will deny. And its application to the case before us is direct and palpable.

The Mississippi Valley has two peculiarities. It is, we believe, the *largest* valley in the world, and, throughout its whole extent, the most strikingly *similar to itself*, in all its *leading features* and *characteristics.* This the Editors of the Examiner will satisfactorily learn, if they will visit and examine it. And the tour would be equally *instructive* and *gratifying* to them. They would then become apprized, that from the Lakes to the Gulf of Mexico, and from the Allegheny to the Rocky Mountains, the *geological formation* of the Valley is the same; a circumstance which has a controlling influence on all its productions. And they would become farther apprized, that the febrile complaints in every section of it are so similar to each other, that a knowledge of them in *any* one part of it, is a knowledge of them in *every* other part. A practical acquaintance with them in the *immediate* valley of the Ohio, where Louisville stands, furnishes an acquaintance with them in every portion of the Mississippi Valley, sufficient for the purposes of medical instruction. This is a position which no western physician of judgment and experience will gainsay or question. Nor will the Editors of the Examiner question it, provided they take the proper steps to inform themselves on the subject. And such steps, when taken, will instruct them on another point, respecting which they need instruction—that the febrile complaints of the West and South are exceedingly dissimilar to those of the "*sea-board*" in the middle and northern States, and require different modes of treatment. In a word; the experiment, if judiciously conducted, will convince them most fully, of the insufficiency of Atlantic Professors to communicate to medical pupils a *practical* knowledge of the febrile diseases of the Mississippi valley. And this is all we have contended for. We have never denied to the Professors of the Atlantic schools a full competency to instruct their pupils in the nature and treatment of Atlantic diseases. But their competency, as relates to western complaints, we do deny, and assert the contrary opinion to be at war with reason, as well as with experience and common sense. As a conclusive experiment on this subject, we challenge any Professor east of the Alleghany mountains, and residing in the middle or northern States, or all of such Professors combined, to write, from their own resources, a book on the treatment of western febrile complaints, and submit it to the judgment of western physicians, who are skilled in those complaints. Let this be done; and should such a book pass such an ordeal uncondemned, we will surrender our opinion on the subject, and acknowledge ourselves vanquished. But, should our challenge be declined, we shall hold ourselves authorized to pronounce such declension an acknowledgment, on the part of the eastern Professors, that they feel themselves incompetent to the enterprise proposed; and that they are therefore afraid to peril their reputation in it. And such acknowledgment will be self-condemnation, as relates to their fitness to communicate, in their lectures, such instruction as will possess any material value, with regard to the treatment of Mississippi fevers. But, say the editors,

"A man of intelligence, who is thoroughly grounded in the elements of medicine, will find no difficulty in adapting general principles to peculiar circumstances."

This sentence is somewhat obscure, if not enigmatical. But it may be thus unriddled:

A physician educated in an eastern school may come to the west, and *here learn by experience how to treat our western* complaints!

True; he may; and many instances of the kind have occurred. But wo! to the patients in whose cases he begins, pursues, and finishes his *practical apprenticeship!* and wo! to the condition of his own mind, if he feel as he ought to do, for the credit of his profession, and for human suffering and loss of life. And if his own reputation do not share in the suffering and deplorable fate of his patients, his lot will be fortunate beyond all reasonable ground of expectation or hope.

Before he can be prepared for the successful performance of his duty as a practitioner, he must accomplish two tasks of weight and difficulty—forget the old and false precepts of practice, which he had imbibed from his eastern preceptors; and derive new and correct ones from observation and experience. On the contrary, the pupil who is educated in a western institution, escapes much of this trial, so *perilous*, not to say *destructive* to the sick, so distressing to himself and so subversive of his reputation. The reason is plain. While he is instructed in the *principles* of his profession, he is schooled also in an acquaintance with correct *practice.*

The exception taken to our review of Professor Hosack's "Lectures" in the "American Medical Library," is of a character altogether different from that in the Examiner. It is a censure, for no other reason, but because the editor is *resolved* to censure, and because, as he alleges, he knows the writer. Assuredly no other reason appears. Nor is the censure open and manly. It is one of those *oblique* strokes, which a man of more *cautiousness* than *courage*, who means to *inflict* an injury, without *receiving* one, *aims in the dark!*

For this reason, although we had determined to notice the attack, perhaps with some severity, and had even in part prepared a few pages to that effect, we shall decline publishing them, because we cannot stoop to engage at present in a contest, to be carried on chiefly by unkind feelings and disrespectful words. Should the editor of the "Medical Library" however continue to repeat his assaults, he may perhaps hereafter be replied to on the subject. We promise him that this shall certainly be the case, if he will *himself* write an article of *any merit*, in opposition to our review of Professor Hosack's "Lectures," and publish it in his Journal. C. C.

